# Prevalence and predictive value of ICD‐11 post‐traumatic stress disorder and Complex PTSD diagnoses in children and adolescents exposed to a single‐event trauma

**DOI:** 10.1111/jcpp.13240

**Published:** 2020-04-28

**Authors:** Rachel Elliott, Anna McKinnon, Clare Dixon, Adrian Boyle, Fionnuala Murphy, Theresa Dahm, Emma Travers‐Hill, Cari‐lène Mul, Sarah‐Jane Archibald, Patrick Smith, Tim Dalgleish, Richard Meiser‐Stedman, Caitlin Hitchcock

**Affiliations:** ^1^ MRC: Cognition and Brain Sciences Unit University of Cambridge Cambridge UK; ^2^ Centre for Emotional Health Macquarie University Sydney NSW Australia; ^3^ Cambridge University Hospitals NHS Foundation Trust Cambridge UK; ^4^ Department of Psychology Anglia Ruskin University Cambridge UK; ^5^ Institute of Psychiatry, Psychology and Neuroscience King's College London London UK; ^6^ Cambridgeshire and Peterborough NHS Foundation Trust Cambridge UK; ^7^ Department of Clinical Psychology Norwich Medical School University of East Anglia Norwich UK

**Keywords:** Post‐traumatic stress disorder, child, adolescent, trauma, Complex PTSD, International Classification of Diseases

## Abstract

**Background:**

The 11th edition of the International Classification of Diseases (ICD‐11) made a number of significant changes to the diagnostic criteria for post‐traumatic stress disorder (PTSD). We sought to determine the prevalence and 3‐month predictive values of the new ICD‐11 PTSD criteria relative to ICD‐10 PTSD, in children and adolescents following a single traumatic event. ICD‐11 also introduced a diagnosis of Complex PTSD (CPTSD), proposed to typically result from prolonged, chronic exposure to traumatic experiences, although the CPTSD diagnostic criteria do not require a repeated experience of trauma. We therefore explored whether children and adolescents demonstrate ICD‐11 CPTSD features following exposure to a single‐incident trauma.

**Method:**

Data were analysed from a prospective cohort study of youth aged 8–17 years who had attended an emergency department following a single trauma. Assessments of PTSD, CPTSD, depressive and anxiety symptoms were performed at two to four weeks (*n* = 226) and nine weeks (*n* = 208) post‐trauma, allowing us to calculate and compare the prevalence and predictive value of ICD‐10 and ICD‐11 PTSD criteria, along with CPTSD. Predictive abilities of different diagnostic thresholds were undertaken using positive/negative predictive values, sensitivity/specificity statistics and logistic regressions.

**Results:**

At Week 9, 15 participants (7%) were identified as experiencing ICD‐11 PTSD, compared to 23 (11%) experiencing ICD‐10 PTSD. There was no significant difference in comorbidity rates between ICD‐10 and ICD‐11 PTSD diagnoses. Ninety per cent of participants with ICD‐11 PTSD also met criteria for at least one CPTSD feature. Five participants met full CPTSD criteria.

**Conclusions:**

Reduced prevalence of PTSD associated with the use of ICD‐11 criteria is likely to reduce identification of PTSD relative to using ICD‐10 criteria but not relative to DSM‐4 and DSM‐5 criteria. Diagnosis of CPTSD is likely to be infrequent following single‐incident trauma.

## Introduction

Accurate diagnosis of trauma‐related difficulties is vital not only to maximise quality of life for affected individuals, but also to ensure appropriate service commission and delivery. In an effort to improve the accuracy of diagnoses, the 11th edition of the International Classification of Diseases (ICD‐11; World Health Organization, 2018) made substantial amendments to the criteria for trauma‐related disorders. In addition to changes to the post‐traumatic stress disorder (PTSD) criteria, an additional disorder was introduced—Complex PTSD (CPTSD), whereby full PTSD diagnostic criteria are met in addition to three additional symptom clusters that reflect disturbances in self‐organisation: affect dysregulation, negative self‐concept, and interpersonal difficulties. Here we seek to evaluate the clinical utility of these new and revised diagnoses for children and adolescents by determining the occurrence and persistence of both ICD‐11 PTSD and CPTSD in young people who attended a hospital following a single traumatic event.

The revised ICD‐11 criteria for PTSD focus on a smaller number of core symptoms. Although the three core PTSD symptom clusters (avoidance, re‐experiencing, hyperarousal) were retained, ICD‐11 omits the symptoms common to both PTSD and other disorders—sleep difficulties and concentration difficulties—in an effort to improve the specificity of the PTSD diagnosis (Maercker et al., 2013). Furthermore, a requirement of functional impairment was introduced into the ICD‐11 criteria (mirroring the Diagnostic and Statistical Manual for Mental Disorders [DSM]) in an effort to differentiate PTSD from normal reactions to extreme stressors and address concerns that the threshold for ICD‐10 PTSD was relatively low (Maercker et al., 2013). There is some evidence that ICD‐11 may reduce diagnostic rates relative to ICD‐10 (e.g. Sachser & Goldbeck, 2016), and DSM‐5 (Sachser et al., 2017), in young people being treated for post‐traumatic stress. ICD‐11 has also been found to reduce diagnosis relative to DSM‐5 in preschool (1–6 year old) children (Vasileva, Haag, Landolt, & Petermann, 2018). Preliminary evidence has suggested that use of ICD‐11 criteria in community‐based samples of young people is unlikely to substantially reduce prevalence rates relative to DSM criteria; however, results have been more mixed in defined samples of trauma survivors (for review see Brewin et al., 2017). Further, the predictive value of the ICD‐11 PTSD diagnosis has not been well explored in young people, which has important implications for whether the revised criteria reduce diagnosis of normal reactions to adverse events which are likely to naturally recover over time (Hiller et al., 2016).

The omission of symptoms common to other disorders may also reduce comorbidities relative to diagnosis using DSM criteria (Maercker et al., 2013). However, by making the ICD‐11 PTSD criteria more stringent, it may also plausibly follow that comorbidity is likely to increase as only the more severe cases will be diagnosed. This is consistent with some findings within the adult literature, whereby ICD‐11 has been associated with higher rates of disability and higher comorbidity with anxiety (but not depression) compared to the diagnosis of PTSD using DSM‐5 (Shevlin et al., 2018). The first aim of this study therefore was to determine if the ICD revision had delivered its intended effects by evaluating whether use of ICD‐11 PTSD criteria (a) reduces the estimated prevalence of PTSD, relative to ICD‐10 criteria, and (b) impacts the estimated frequency of comorbid difficulties in youth. The specificity of the ICD‐11 PTSD diagnosis is particularly important to explore in children and adolescents, as the behavioural symptoms that were removed are markers of PTSD that are more readily identifiable by parents and caregivers compared to children's cognitive symptoms which are harder to evaluate by observers (for review, see Smith et al., 2018).

The ICD‐11 also presents different diagnoses for PTSD and Complex PTSD (CPTSD). The introduction of CPTSD criteria follows clinical observations (Cloitre, et al., 2013; Herman, 1992) that individuals exposed to very severe, repeated or prolonged traumas often demonstrate additional disturbances in self‐organisation that impact functioning and require treatment (Shevlin et al., 2018). In addition to the three core features of PTSD (re‐experiencing, avoidance and hyperarousal), diagnosis of CPTSD requires one symptom from each of three additional symptom clusters—affect dysregulation, negative self‐concept and interpersonal difficulties. Although originally conceptualised as more likely to occur in those with complex trauma histories (Van der Kolk, Roth, Pelcovitz, Sunday, & Spinazzola, 2005), the ICD‐11 CPTSD criteria do not include a requirement for repeated or prolonged traumatic events thus acknowledging the possibility of more complex presentations following single‐event traumas.

Prior latent class analyses have endorsed distinct symptom profiles for PTSD vs CPTSD in both community (Perkonigg et al., 2016) and treatment‐seeking (Sachser et al., 2017) child and adolescent samples. However, no prior study has explored either natural recovery from, or persistence of, CPTSD features, or the impact of trauma frequency (particularly single‐incident events) on children's experience of CPTSD symptoms. Childhood and adolescence is a developmentally sensitive period for the self‐organisation structures that become distorted in CPTSD (i.e. interpersonal skills, self‐identity and affect regulation). As such, it is plausible that a single traumatic incident during this developmental window may be sufficient to impair these structures and produce complex post‐traumatic stress responses in youth. If so, this is important to determine given that the more Complex PTSD presentations are associated with greater functional impairments (Stein et al., 1997) and put individuals at risk of misdiagnosis (Herman, 1992). Thus, the second aim of this study was to explore whether children and adolescents might demonstrate CPTSD features following a single‐incident trauma. Importantly, we examined how the presence of CPTSD features related to comorbid depression and anxiety difficulties, and pretrauma concerns for the young person's mental health.

To address these aims, we analysed data collected from 8–17 years old (see Meiser‐Stedman et al., 2017), who presented at one of four hospital emergency departments following a single‐incident traumatic event. Assessments were completed at two weeks and nine weeks following the traumatic event, which allowed us to calculate and compare the prevalence, specificity and predictive value of ICD‐10 and ICD‐11 PTSD criteria^1^, along with CPTSD.

## Method

### Participants

Participants (aged 8–17 years, *M* = 14.1 years, *SD* = 7.2) and their caregivers were recruited at four emergency departments in the East of England following a single‐event trauma between September 2010 and April 2013. In this data set, traumatic events had been defined using the DSM‐5 definition of a trauma, that is involving the threat of death or serious injury, or as an event that lead to admission to intensive care, high dependency unit or admission for three or more days to hospital. A trauma was considered a single event if it was a ‘one‐off’ incident unrelated to maltreatment or abuse. Exclusion criteria were inability to speak English, intellectual disability, attendance resulting from deliberate self‐harm, being under the care of child protection services and moderate to severe traumatic brain injury (i.e. post‐traumatic amnesia ≥24 hr).

Two hundred and twenty‐six (37.4%) of the 604 eligible participants completed the initial Week 2 assessment (days since trauma, *M* = 22.0, *SD* = 7.2). Ninety‐six of these participants (42.5%) were female and sixteen (7.1%) belonged to a minority ethnic group or were mixed race. Approximately 16% had received treatment for a mental health issue prior to the trauma. There were no significant differences between participants and eligible nonparticipants in terms of age, sex, ethnicity, number of injuries, having a medical procedure in the Emergency Department, being seen in the resuscitation area of the Emergency Department, days admitted, previous attendances, head injury or Glasgow Coma Scale scores in those with mild traumatic brain injury (*p*s > .05).

Two hundred and eight participants (92.0% of those who completed the Week 2 assessment) completed a second assessment 9 weeks post‐trauma (*M* = 67.5 days, *SD* = 11.7). There were no differences between youth who did or did not complete the Week 9 assessment in terms of sex, age or initial traumatic stress symptoms (*p*s *>* .15).

### Trauma and injury characteristics

Participants had experienced a motor vehicle collision (*n* = 104; 46.0%), an assault (*n* = 41; 18.1%), a dog attack (*n* = 10; 4.4%), serious accidental injuries (*n* = 70; 31.0%) and a medical emergency (*n* = 1; 0.4%). Forty‐eight (21.2%) sustained a bone fracture, 62 (27.4%) were admitted to hospital and 13 (5.8%) to an intensive care unit. Thirty‐nine (17.3%) received opiate medication. Eighty‐six (38.1%) sustained a head injury during the trauma, and of these, 26 (11.5%) lost consciousness during or shortly after the trauma. Nine (4.0%) were intubated at the scene of the trauma.

### Measures

#### PTSD and CPTSD diagnosis

The self‐report items that we used to index ICD‐11 diagnostic criteria for PTSD and CPTSD are presented in Table S1. Diagnosis of PTSD requires the presence of one of two items in the re‐experiencing, avoidance and hyperarousal clusters, respectively, along with functional impairment as a result of these symptoms. In addition to presence of these criterion, CPTSD requires one of two items in the affective dysregulation, negative self‐beliefs and interpersonal difficulties cluster, along with functional impairment.

The International Trauma Questionnaire is the leading self‐report measure of ICD‐11 PTSD and CPTSD (Cloitre et al., 2018) and has been recently validated for use in children and young people (Haselgruber et al., 2019). As we made use of secondary data that were collected prior to publication of the ITQ, we sought to match the ITQ items with participants' responses (see Table S1) on the Child PTSD Symptom Scale (CPSS, Foa, Johnson, Feeny, & Treadwell, 2001), the Child Post‐Traumatic Cognitions Inventory (CPTCI, Meiser‐Stedman et al., 2009) and the Child Response Style Questionnaire (CRSQ, Meiser‐Stedman et al., 2017). This ensured that our measurement of ICD‐11 PTSD and CPTSD was comparable to the wider literature. In aid of this, our chosen items were also matched to the ITQ items based on the methods of previous studies. For example, for the ITQ item ‘*When I am upset, it takes me a long time to calm down*’, we used the CPSS item ‘*Feeling irritable and having fits of anger*’, based on Perkonigg et al. (2016) and Sachser et al.'s (2017) use of self‐report of anger and irritability to index this criterion.

We rated a diagnostic criterion as met if the participant’s response on the matched self‐report item indicated that the symptom was present ‘2–4 times a week/half the time’ or ‘5 or more times a week/all the time’ (i.e. a score of 2 or above, out of 3) on the CPSS, and ‘agree a bit’ or ‘agree a lot’ (i.e. a score of 3 or above, out of 4) for CPTCI items (as per Sachser et al., 2017). This accords with ITQ scoring criteria (Cloitre et al., 2018). For the functional impairment criterion to be met, participants needed to respond ‘Yes’ to one of the CPSS Yes or No questions asking whether their symptoms had got in the way of relationships with friends, relationship with family, schoolwork, fun and hobby activities, or chores and duties at home. For example, the ITQ item ‘*Affected your relationships or social life*?’ was matched as reporting yes to either of the CPSS functional impairment items ‘*Has the problem got in the way of your relationships with friends*’ or ‘*Has the problem got in the way of your relationships with your family*’. Cronbach’s alpha was acceptable for both scales ICD‐11 PTSD = .85; ICD‐11 CPTSD = .74.

Using this scoring procedure, ICD‐11 PTSD and CPTSD diagnoses were scored at Week 9, and satisfying the nonduration PTSD criteria was scored at Week 2. We report DSM diagnoses from the original paper (Meiser‐Stedman et al., 2017) for comparison purposes. Diagnosis of ICD‐10 PTSD followed the above ICD‐11 procedure. Items used to index ICD‐10 PTSD diagnostic criteria are presented in Table S2. Cronbach's α was good, ICD‐10 PTSD = .87.

#### Comorbid difficulties

Youth with clinically significant anxiety and depression were identified using self‐report questionnaire cut‐offs for the 38‐item Spence Child Anxiety Scale (scores ≥ 60 indicate clinically significant anxiety; possible range 0–114) and the 13‐item Short Mood and Feelings Questionnaire (scores ≥ 8 indicate clinically significant depression; possible range 0–26), respectively (Costello & Angold, 1988; Spence, 1998).

### Procedure

Data collection was approved by the UK National Research Ethics Service, Cambridgeshire Research Ethics Committee (10/H0304/11). The caregivers of children meeting inclusion criteria were initially contacted by letter 2–4 days post‐hospital attendance and then by telephone at 7–8 days to arrange the initial Week 2 assessment. At Week 2, caregivers answered additional questions about their child’s trauma and subsequent hospital attendance. Further information (e.g. extent of injuries) was obtained from medical records. Follow‐up assessments were completed nine weeks post‐trauma. For full details, see Meiser‐Stedman et al. (2017).

### Statistical analyses

Prevalence of youth meeting criteria for PTSD and CPTSD (minus the duration criterion) at Week 2 was calculated using the entire sample. Predictive utilities of different diagnostic thresholds were examined using positive/negative predictive values, sensitivity/specificity statistics and logistic regressions. For these analyses, only the cases (*n* = 208) with full Week 2 and Week 9 data were included.

Under ICD‐11 criteria, diagnosis of CPTSD requires that ICD‐11 PTSD criteria are met first. Although presence of CPTSD is listed as an exclusion for ICD‐11 PTSD in the ICD‐11 diagnostic criteria, it is unlikely that CPTSD will be routinely assessed in young people exposed to a single‐incident trauma. Thus, to ensure that prevalence of ICD‐11 PTSD in this sample was not under‐represented in comparisons with ICD‐10 PTSD, cases that went on to meet CPTSD criteria were included when completing between‐group comparisons of prevalence and comorbidity between ICD‐10 and ICD‐11 PTSD. The prevalence of ICD‐11 PTSD when excluding cases with CPTSD is reported in Table 1. Positive/negative predictive values and sensitivity/specificity statistics for ICD‐11 PTSD excluded those cases who met CPTSD criteria.

## Results

### PTSD prevalence

The prevalence of those meeting ICD‐10 and ICD‐11 PTSD criteria (minus the duration criterion) at Week 2 and qualifying for PTSD at Week 9, along with data on comorbid problems, is presented in Table 1. McNemar’s tests indicated that ICD‐11 diagnostic rates did not significantly differ from DSM‐IV or DSM‐5 at either Week 2, *p*s > .06, or Week 9, *p*s > .21. However, ICD‐10 PTSD criteria were endorsed significantly more than ICD‐11 at both Week 2, *p* < .001, and Week 9, *p* = .008.

**Table 1:** Prevalence of PTSD, CPTSD and elevated symptoms of comorbid difficulties at Week 2 (minus PTSD duration criteria) and Week 9

	Week 2 (*n* = 226)		Week 9 (*n* = 208)	
	*n* (% of total sample)	% of those meeting the specified PTSD criteria (minus duration)	*n* (% of total sample)	% of those meeting the specified PTSD criteria
DSM‐IV	40 (17.7)	–	18 (8.7)	–
DSM‐5	41 (18.1)	–	20 (9.6)	–
ICD‐10 PTSD	44 (19.5)	–	23 (11.1)	–
With comorbid anxiety	11 (4.8)	25.0	4 (1.9)	17.4
With comorbid depression	32 (14.2)	72.7	13 (6.3)	56.5
All ICD‐11 PTSD cases	26 (11.5)	–	15 (7.2)	–
With comorbid anxiety	9 (4.0)	34.6	2 (1.0)	13.3
With comorbid depression	22 (9.7)	84.6	8 (3.8)	53.3
ICD‐11 PTSD (excluding CPTSD cases)	15 (6.6)	–	10 (4.8)	–
with comorbid anxiety	2 (0.90.9)	13.3	1 (0.5)	10.0
with	11 (4.9)	73.3	3 (1.4)	30.0
ICD‐11 PTSD with at least 1 Complex feature	12 (5.3)	80.0	9 (4.3)	90.0
Affect regulation	9 (4.0)	60.0	6 (2.9)	60.0
Negative beliefs	3 (1.3)	20.0	3 (1.4)	30.0
Interpersonal difficulties	9 (4.0)	60.0	6 (2.9)	60.0
ICD‐11 PTSD with 2 Complex features	9 (4.0)	60.0	6 (2.9)	60.0
CPTSD	11 (4.8)	–	5 (2.4)	–
With comorbid anxiety	7 (3.1)	63.6	1 (0.5)	20.0
With comorbid depression	11 (4.8)	100.0	5 (2.4)	100.0

For comparison purposes, DSM‐IV and DSM‐5 rates are presented (from Meiser‐Stedman et al., 2017). Comorbid depression was indexed as a score ≥ 8 on the Short Mood and Feelings Questionnaire; comorbid anxiety was indexed as a score of ≥ 60 on the Spence Child Anxiety Scale. The number ‘with comorbid depression’ or ‘with comorbid anxiety’ is the number of participants with the given PTSD diagnosis who also had comorbid anxiety or depression. Week 2 ICD‐10 and ICD‐11 PTSD rates ignore the duration requirement for diagnosis. Frequency of 1 and 2 complex features excludes those who met CPTSD criteria.

At Week 9, all children diagnosed with PTSD using the ICD‐11 also met ICD‐10 criteria for diagnosis. The mean for self‐report symptoms on the CPSS was similar when using ICD‐11 (*M* = 29.67, *SD* = 7.50, range = 2–39) compared to ICD‐10 (*M* = 27.65, *SD* = 8.42, range = 2–39). This meant that eight of the children (35%) who met criteria for PTSD according to the ICD‐10 did not receive a diagnosis according to the ICD‐11 criteria. The mean self‐reported symptom level in those who met ICD‐10 but not ICD‐11 diagnostic criteria was above (16) the clinical cut‐off for significant distress and impairment on the CPSS (*M* = 23.88, *SD* = 9.23). Specifically, 6 children (75%) diagnosed with ICD‐10 but not ICD‐11 PTSD scored above the CPSS clinical cut‐off. There were no systematic differences in age (*p* = .508) or sex (*p* = .980) between those children meeting criteria for ICD‐10 and ICD‐11 PTSD, relative to those who met ICD‐10 criteria alone. In terms of specific symptom clusters, of all those who met full ICD‐10 criteria, 91% met the ICD‐11 hyperarousal cluster, 78% met the re‐experiencing cluster, and 100% met the avoidance cluster. A key difference between the ICD‐10 and ICD‐11 PTSD diagnoses is the requirement of impairment; only 87% of participants diagnosed with the ICD‐10 met the ICD‐11 impairment requirement.

At Week 2, McNemar’s tests indicated a significantly greater percentage of those with ICD‐11 PTSD also experienced comorbid depression (84.6%), relative to those diagnosed using ICD‐10 PTSD criteria (72.7%), *p *= .02. At Week 9, there was no significant difference in rates of comorbid depression or anxiety between those diagnosed using ICD‐10 or ICD‐11 PTSD criteria, *p*s > .06.

Re‐analysis of ICD‐10 vs ICD‐11 prevalence and comorbidity indicated similar results when CPTSD cases were excluded from ICD‐11 PTSD, with the exception that the Week 9 difference in rates of comorbid depression became significant, *p* = .008.

### How well does satisfying PTSD criteria at Week 2 predict PTSD diagnosis at Week 9?

The degree to which satisfying the PTSD criteria (minus duration) at Week 2 can predict PTSD at Week 9 is detailed in Table 2. The number of participants meeting PTSD criteria roughly halved between the two assessments, regardless of the ICD version used. Satisfying either ICD‐10 or ICD‐11 Week 2 PTSD criteria was significantly predictive of later PTSD diagnoses, as indexed by regression statistics. Each ICD version yielded high negative predictive values (all =.97), but positive predictive values were modest.

**Table 2:** Predictive statistics for ICD PTSD diagnoses

Week 2 Predictor (ignoring duration criterion)	Regression statistics			Positive predictive value	Negative predictive value	Sensitivity	Specificity	Percentage correctly identified
	χ^2^	*p*	Odds ratio					
ICD‐10 PTSD criteria	49.99	<.001	22.35	0.43	0.97	0.78	0.86	0.88
ICD‐11 PTSD criteria	42.42	<.001	22.13	0.40	0.97	0.67	0.92	0.92

Cases of CPTSD are not included in this analysis. Positive predictive value = Likelihood that someone meeting criteria at Week 2 would have the relevant diagnosis at Week 9. Negative predictive value = Likelihood that someone not meeting criteria at Week 2 would not go on to have the relevant diagnosis at Week 9. Sensitivity = Likelihood that someone with a diagnosis at Week 9 would have previously met criteria for at Week 2. Specificity = Likelihood that someone without diagnosis at Week 9 would not have met criteria at Week 2.

### What is the prevalence of Complex PTSD features?

The prevalence rates for CPTSD features are presented in Table 1. At Week 9, five participants met full diagnostic criteria for CPTSD. All five individuals also scored above the clinical cut‐off on the depression measure, and one scored above the cut‐off for anxiety. Four of the five had a prior history of trauma and four of the five had experienced mental health concerns prior to the traumatic event indexed in this study.

Complex features were also evident in those who did not meet full CPTSD criteria. Ninety per cent of participants who met ICD‐11 PTSD criteria (but not CPTSD) at Week 9 also additionally endorsed one or two (out of three) of the CPTSD clusters. Of the nine participants demonstrating one CPTSD feature, one experienced comorbid anxiety (11%) and three experienced comorbid depression (30%). Of the six participants demonstrating two CPTSD features, one experienced comorbid anxiety (20%), two experienced comorbid depression (40%), and one had missing comorbidity data.

## Discussion

This study sought to evaluate the prevalence, specificity and predictive value of ICD‐11 PTSD and CPTSD diagnoses in children and adolescents aged 8–17 years who attended hospital following a single‐incident trauma. As anticipated, the revised ICD‐11 PTSD diagnosis was more clinically conservative than the ICD‐10 algorithm and did slightly improve the specificity of diagnosis. A lower percentage of participants diagnosed using ICD‐11 appeared to experience comorbid difficulties compared to ICD‐10, but these rates were only statistically different when CPTSD cases were excluded. Results also indicated that the incidence of CPTSD following a single‐incident trauma was infrequent and predominately occurred in combination with other mental health concerns, most notably depression, and a prior history of trauma. However, complex features of PTSD were evident in some children and adolescents as early as two weeks following a single‐incident trauma, and the vast majority of participants diagnosed with ICD‐11 PTSD (but not CPTSD) at Week 9 also experienced one feature of ICD‐11 CPTSD. It is important to note that approximately half of those identified by ICD‐11 PTSD also experienced elevated depression and/or anxiety symptoms, which may impact identification of CPTSD symptoms. These findings suggest that repeated or prolonged trauma may not be necessary to induce complex post‐traumatic stress features in young people. However, further exploration of the relationship between CPTSD features, pretrauma mental health concerns, trauma history and comorbid difficulties is needed, and diagnosis of CPTSD is likely to be uncommon following a single‐incident trauma.

The majority of participants did not satisfy PTSD criteria at either assessment, and there was considerable reduction in those satisfying criteria between assessments, corroborating previous research showing considerable natural recovery in youth exposed to trauma (Hiller et al., 2016). Although diagnostic rates were similar between DSM classifications and ICD‐11 (consistent with Danzi & La Greca, 2016; Hafstad et al., 2017), diagnostic rates for PTSD varied significantly between the different ICD diagnostic versions. Our findings were consistent with the stated aim for the ICD‐11 to reduce diagnostic rates of PTSD, relative to ICD‐10. At Week 9, the application of ICD‐11 PTSD criteria reduced the diagnostic prevalence rates by approximately 5%, relative to applying the ICD‐10 criteria. Use of the ICD‐11 criteria was also associated with improved specificity of diagnosis, relative to use of the ICD‐10 criteria. The mean self‐reported symptoms in those children diagnosed with ICD‐10 PTSD but not meeting ICD‐11 criteria were however still above the clinical cut‐off for significant distress and functional impairment. As such, if transitioning from use of ICD‐10 to ICD‐11 diagnostic criteria as a requirement for access to treatment, it is important to consider that young people with subthreshold ICD‐11 symptoms may be experiencing significant distress and may still be in need of treatment to reduce the detrimental longer‐term impact of post‐traumatic stress during these developmental years. Further exploration of whether such children may receive treatment for other mental health issues (e.g. depression, anxiety and conduct) is needed.

Our results suggest that diagnosis of CPTSD is uncommon in young people exposed to a single‐incident trauma. However, the majority of participants diagnosed with PTSD also met criteria for one of the three disorganised self‐structure symptoms required for a CPTSD diagnosis. Thus, although a diagnosis of CPTSD may be infrequent following single trauma exposure, our results do suggest that assessment of CPTSD features, most notably affect dysregulation and interpersonal difficulties (along with comorbid depression and anxiety), may be warranted in young people following a single‐incident trauma, including noninterpersonal accidental traumas. Importantly, our results suggest that complex features are not solely attributable to comorbid anxiety or depression, as the majority of youth who endorsed one CPTSD cluster did not demonstrate comorbid difficulties.

There are a number of limitations to this study. First, analysis of secondary data ensures that data collected from young people and their families are used as comprehensively as possible. However, this did mean that we were required to match data collected on validated self‐report measures as closely as possible to the ITQ, the most prevalent self‐report measure of ICD‐11 PTSD and CPTSD. Similarly, participants had been recruited on the basis of meeting the DSM criterion for a traumatic event, rather than the ICD guidance regarding potential events which may cause PTSD. As we relied on self‐report measures of comorbid depression and anxiety symptoms, future research will need to explore formal comorbid diagnoses against relevant ICD‐11 criteria, in studies with greater power, to strengthen our conclusions. Second, some of our analyses were limited to complete cases across time points. Our sample size at Week 9 was relatively modest and thereby likely underpowered compared to evaluation of ICD criteria in adult samples (e.g. Barbano et al., 2019), as is common in PTSD research with younger samples.

Future evaluation of the ICD criteria with a larger sample of young people with more varied trauma experiences, namely single‐incident events that did not result in hospital attendance, as well as those who have experienced multiple or repeated traumas, will allow more specific evaluation of the presence of complex features following different trauma types (e.g. those that are interpersonal in nature vs. those that are not). However, this study has taken important steps towards improving accurate identification of post‐traumatic stress disorders in young people by demonstrating that ICD‐11 criteria are likely to reduce diagnosis of PTSD relative to ICD‐10, just as it does in adults. Further, we have emphasised the possibility that young people may appear to experience some Complex PTSD features following a single‐incident event, but few are likely to meet CPTSD diagnostic criteria.

## Supporting information


**Table S1.** Matching of International Trauma Questionnaire items to index ICD‐11 PTSD and Complex PTSD criteria.
**Table S2.** Matching of Child Posttraumatic Stress Scale (CPSS) items to index ICD‐10 PTSD criteria.

## Note

1 At the two‐week assessment, a formal diagnosis of PTSD was not possible as the duration criterion of 1 month cannot be met. We therefore examined the predictive utility of satisfying all of the other PTSD criteria (minus duration) at this time point, according to ICD‐10 and ICD‐11. Henceforth, we refer to this as satisfying the 2‐week (C)PTSD criteria to reflect these parameters.
