# Supplementary material for: Prevalence and predictive value of ICD‐11 post‐traumatic stress disorder and Complex PTSD diagnoses in children and adolescents exposed to a single‐event trauma
Source: J Child Psychol Psychiatry. 2020 Apr 28;62(3):270–6. doi: 10.1111/jcpp.13240 (PMC7984249; doi:10.1111/jcpp.13240)
Supplement: Supplementary file 1 — Table S1. Matching of International Trauma Questionnaire items to index ICD‐11 PTSD and Complex PTSD criteria. Table S2. Matching of Child Posttraumatic Stress Scale (CPSS) items to index ICD‐10 PTSD criteria. [file JCPP-62-270-s001.docx]

**Supporting information – Prevalence and predictive value of ICD-11 posttraumatic stress disorder (PTSD) and Complex PTSD diagnoses in children and adolescents exposed to a single-event trauma – by Elliott *et al*.**

**Table S1.** Matching of International Trauma Questionnaire items to index ICD-11 PTSD and Complex PTSD criteria.

| ITQ item | Matched item |
| --- | --- |
| P1  Having upsetting dreams that replay part of the experience or are clearly related to the experience? | CPSS 2  Having bad dreams or nightmares |
| P2  Having powerful images or memories that sometimes come into your mind in which you feel the experience is happening again in the here and now? | CPSS 3  Acting or feeling as if the event was happening again (hearing something or seeing a picture about it and feeling as if I am there again) |
| P3  Avoiding internal reminders of the experience (for example, thoughts, feelings, or physical sensations)? | CPSS 6  Trying not to think about, talk about, or have feelings about the event |
| P4  Avoiding external reminders of the experience (for example, people, places, conversations, objects, activities, or situations)? | CPSS 7  Trying to avoid activities, people, or places that remind you of the traumatic event |
| P5  Being “super-alert”, watchful, or on guard? | CPSS 16  Being overly careful (for example, checking to see who is around you and what is around you) |
| P6  Feeling jumpy or easily startled? | CPSS 17  Being jumpy or easily startled (for example, when someone walks up behind you) |
| P7  Affected your relationships or social life | CPSS 23  Affected your relationships with friends  CPSS 25  Affected your relationships with your family |
| P8  Affected your work or ability to work? | CPSS 24  Affected your schoolwork |
| P9  Affected any other important part of your life such as parenting, or school or college work, or other important activities? | CPSS 22  Affected your fun and hobby activities  CPSS 26  Affected your chores and duties at home |

| ITQ item | Matched item |
| --- | --- |
| C1  When I am upset, it takes me a long time to calm down | CPSS 14  Feeling irritable and having fits of anger |
| C2  I feel numb or emotionally shut down | CPSS 11  Not being able to have strong feelings (for example, being unable to cry or unable to feel happy) |
| C3  I feel like a failure. | CPTCI 8  Not being able to get over all my fears means that I am a failure |
| C4  I feel worthless. | CPTCI 7  I am no good |
| C5  I feel distant or cut-off from people. | CPSS 10  Not feeling close to people around you |
| C6  I find it hard to stay emotionally close to people | CPTCI 5  I don’t trust other people |
| C7  Created concern or distress about your relationships or social life? | CPSS 23  Affected your relationships with friends  CPSS 25  Affected your relationships with your family |
| C8  Affected your work or ability to work? | CPSS 24  Affected your schoolwork |
| C9  Affected any other important parts of your life such as parenting, or school or college work, or other important activities? | CPSS 22  Affected your fun and hobby activities  CPSS 26  Affected your chores and duties at home |

*Note.* ITQ=International Trauma Questionnaire; CPSS=Child Posttraumatic Stress Scale; CPTCI=Child Posttraumatic Cognitions Inventory.

**Table S2.** Matching of Child Posttraumatic Stress Scale (CPSS) items to index ICD-10 PTSD criteria.

| ICD-10 | Matched item |
| --- | --- |
| B. Persistent remembering or "reliving" the stressor by intrusive flash backs, vivid memories, recurring dreams, or by experiencing distress when exposed to circumstances resembling or associated with the stressor. | CPSS 1  Having upsetting thoughts or images about the event that came into your head when you didn’t want them  *or*  CPSS 2  Having bad dreams or nightmares  *or*  CPSS 3  Acting or feeling as if the event was happening again (hearing something or seeing a picture about it and feeling as if I am there again)  *or*  CPSS 4  Feeling upset when you think or hear about the event (for example, feeling scared, angry, sad, guilty etc)  *or*  CPSS 5  Having feelings in your body when you think about or hear about the event (for example, breaking out in a sweat, heart beating fast) |
| C. Actual or preferred avoidance of circumstances resembling or associated with the stressor (not present before exposure to the stressor). | CPSS 6  Trying not to think about, talk about, or have feelings about the event  *or*  CPSS 7  Trying to avoid activities, people, or places that remind you of the traumatic event |
| D. Either D.1. *or* D.2.  D.1. Inability to recall, either partially or completely, some important aspects of the period of exposure to the stressor | CPSS 8  Not being able to remember an important part of the upsetting event |
| D.2. Persistent symptoms of increased psychological sensitivity and arousal (not present before exposure to the stressor) shown by any two of the following:   1. difficulty in falling or staying asleep; | CPSS 13  Having trouble falling or staying asleep |
| 1. irritability or outbursts of anger; | CPSS 14  Feeling irritable or having fits of anger. |
| 1. difficulty in concentrating; | CPSS 15  Having trouble concentrating (for example, losing track of a story on television, forgetting what you read, not paying attention in class). |
| 1. hyper-vigilance; | CPSS 16  Being overly careful (for example, checking to see who is around you and what is around you) |
| 1. exaggerated startle response. | CPSS 17  Being jumpy or easily startled (for example, when someone walks up behind you) |

*Note.* CPSS=Child Posttraumatic Stress Scale. ICD-10 PTSD criteria taken from World Health Organization. International statistical classification of diseases and related health problems. 10th revision ed. Geneva, Switzerland: World Health Organization; 1992.
